# Efficient gene correction of an aberrant splice site in β‐thalassaemia iPSCs by CRISPR/Cas9 and single‐strand oligodeoxynucleotides

**DOI:** 10.1111/jcmm.14669

**Published:** 2019-10-21

**Authors:** Zeyu Xiong, Yingjun Xie, Yi Yang, Yanting Xue, Ding Wang, Shouheng Lin, Diyu Chen, Dian Lu, Lina He, Bing Song, Yinghong Yang, Xiaofang Sun

**Affiliations:** ^1^ Key Laboratory for Major Obstetric Diseases of Guangdong Province Key Laboratory of Reproduction and Genetics of Guangdong Higher Education Institutes The Third Affiliated Hospital of Guangzhou Medical University Guangzhou Guangdong China

**Keywords:** CRISPR/Cas9, gene correction, HBB gene, induced pluripotent stem cells, secondary cleavage, single‐stranded oligodeoxynucleotide, β‐Thalassaemia

## Abstract

β‐thalassaemia is a prevalent hereditary haematological disease caused by mutations in the human haemoglobin β (HBB) gene. Among them, the HBB IVS2‐654 (C > T) mutation, which is in the intron, creates an aberrant splicing site. Bone marrow transplantation for curing β‐thalassaemia is limited due to the lack of matched donors. The clustered regularly interspaced short palindromic repeats (CRISPR)/CRISPR‐associated protein 9 (Cas9), as a widely used tool for gene editing, is able to target specific sequence and create double‐strand break (DSB), which can be combined with the single‐stranded oligodeoxynucleotide (ssODN) to correct mutations. In this study, according to two different strategies, the HBB IVS2‐654 mutation was seamlessly corrected in iPSCs by CRISPR/Cas9 system and ssODN. To reduce the occurrence of secondary cleavage, a more efficient strategy was adopted. The corrected iPSCs kept pluripotency and genome stability. Moreover, they could differentiate normally. Through CRISPR/Cas9 system and ssODN, our study provides improved strategies for gene correction of β‐Thalassaemia, and the expression of the HBB gene can be restored, which can be used for gene therapy in the future.

## 1 INTRODUCTION

β‐Thalassaemia, a common genetic disorder, which widely extends from the Mediterranean area, through the Middle East and to Southeast Asia, brings about severe anaemia and skeletal abnormalities. It leads to frequent transfusions and iron chelation therapy for patients.^1^ β‐thalassaemia was caused by the imbalance of globin chain production resulting from gene mutation.^2^ To date, more than 200 mutations from the HBB gene have been identified in the three exons, splicing sites and other regulatory elements. The HBB IVS2‐654 (C > T) mutation, which creates an aberrant splicing site, makes the retention of nucleotides in the second intron and affects normal mRNA transcription.^3^ Bone marrow transplantation (BMT) is the only clinical approach for the cure of β‐Thalassaemia. However, it is still limited by the graft‐vs‐host disease and the paucity of immunologically matched donors.^4^, ^5^ Due to β‐thalassaemia being caused by mutations, gene therapy provides a potential treatment for the disorder.

With the advent of technological breakthroughs on gene editing, the efficient means of curing the genetic disease is to correct the mutation directly via sequence‐specific endonucleases. Endonucleases can create double‐strand breaks (DSBs), which then activates DNA repair by two highly conserved competing mechanisms: nonhomologous end joining (NHEJ) or homology‐directed repair (HDR).^6^, ^7^ NHEJ repairs breaks via ligation of DNA ends throughout the cell cycle, and it causes nearly random insertion and deletion mutations. Nevertheless, HDR can be exploited to make the desired sequence replacement at the DSB site by homologous recombination with a donor DNA template, which is normally most active during the S or G2 phase of the cell cycle. This allows us to utilize HDR to generate targeted gene deletion, mutagenesis, insertion or gene correction.^8^, ^9^

Recently, the clustered regularly interspaced short palindromic repeats (CRISPR)/ CRISPR‐associated protein 9 (Cas9), an RNA‐guided nuclease from an adaptive immune mechanism present in many bacteria and the majority of characterized Archaea, has been widely used as an endonuclease for gene correction through HDR. The system can bind DNA through 20‐bp gRNA which is adjacent to protospacer adjacent motif (PAM) by Watson‐Crick base pairing and then generate the cleavage by Cas9 protein.^10^, ^11^ Due to the fact that CRISPR/Cas9 system is easier to design and construct, as well as having high efficiency of gene editing, many studies have reported using the system for correcting disease‐related mutations in animal somatic^12^ and germ line cells,^13^, ^14^ as well as in human stem cells^15^ and induced pluripotent stem cells.^16^, ^17^ The single‐stranded oligodeoxynucleotide (ssODN) has been applied as the repair template to generate point mutation.^18^ Contrasting to dsDNA, ssODN can be synthesized more easily and quickly, which is not required to excise the section marker. In addition, it was reported that ssODN is more efficient for HDR.^19^

In this study, combining CRISPR/Cas9 and ssODN, we successfully repaired the biallelic HBB IVS2‐654 mutation in induced pluripotent stem cells (iPSCs) via a seamless approach in one step. Because gRNA was not designed at the mutant site resulting from the PAM region‐NGG while the mutation is in intron 2, we could not make a synonymous mutation; a more efficient strategy was adopted to correct the mutation by reducing the occurrence of secondary cleavage. After gene correction, iPSCs still kept pluripotency and genome stability. The corrected iPSCs could be utilized for hematopoietic differentiation normally, and the expression of the HBB gene was restored. Therefore, our study offered improved strategies for gene correction of β‐Thalassaemia.

## 2 MATERIALS AND METHODS

### 2.1 Cell culture and hematopoietic differentiation

The iPSCs derived from a patient with β‐thalassaemia were provided by the Third Affiliated Hospital of Guangzhou Medical University where the experiments were consented by the ethics committee. Human iPSCs were cultured in mTeSR1 medium (Stem Cell) on Matrigel‐coated (Corning) 35 mm dish and passaged with the ratio 1:3‐1:5 every 3‐6 days via the hiPS cell dissociation solution (Cellapy). We replaced new medium daily. The iPSCs with 80% confluence in 35 mm dish were treated with 1 mg/mL dispase (Gibco), and then, small scraped clumps were harvested, which were cultivated on Matrigel‐coated 12‐well plate with 1:60 dilution. According to a five‐step hematopoietic differentiation strategy, the cells were expanded in different mediums containing different cytokines (PeproTech) as previously reported.^20^ At days 12 and 22, differentiated cells were collected via fluorescence‐activated cell sorting (FACS).

### 2.2 Vector construction and gene correction

Guide RNA was designed on the basis of the PAM 5′‐NGG‐3′ for SpCas9, which was inserted into pCAGmCherry‐gRNA vector (Addgene 87110) using Gibson assembly (NEB) following the hCRISPR gRNA synthesis protocol (https://media.addgene.org/data/93/40/adf4a4fe-5e77-11e2-9c30-003048dd6500.pdf). Approximately 2 × 10^6^ iPSCs were treated with Accutase and then electroporated undergoing 1200 V by Neon transfection system (Invitrogen) mixing 2.5 μg gRNA vector, 2.5 μg Cas9 plasmid (Addgene 44719) and 5 μg ssODN (IGE). The transfected iPSCs were plated on Matrigel‐coated 35 mm dish and cultured in mTeSR medium supplemented with 10 μm Y‐27632 (Sigma) for one day. At 48 hours after electroporation, the positive single cells were harvested through FASC (BD Aria III) resulting from gRNA with mCherry reporter, Cas9 with GFP reporter, and replated on Matrigel‐coated 35 mm dish at the density of 5 × 10^3^ cells per dish with 10 μm Y‐27632 for one day. At day 10, we picked part of every clone as DNA template, which were amplified by Pfu PCR MasterMix (Tiangen) and HBB primers. We identified these clones through the products from polymerase chain reaction (PCR) used for Sanger sequencing.

### 2.3 Quantitative polymerase chain reaction analysis

Total RNA from iPSCs was extracted using Quick‐RNA MiniPrep Plus Kit (Zymo Research), and total RNA from differentiated hematopoietic cells at day 22 was isolated via RNeasy Micro Kit (Qiagen) following the manufacturer's instructions. We acquired cDNA utilizing PrimeScript RT reagent Kit with gDNA Eraser (Takara) according to the manufacturer's instructions. Quantitative PCR was performed by StepOnePlus Real‐time PCR system (Applied Biosystems) with SYBR Premix Ex Taq II Til RNaseH Plus (Takara). And *β‐Actin* was used as an internal standard for normalization.

### 2.4 Immunofluorescent staining and alkaline phosphatase staining

Cells were fixed via 4% paraformaldehyde (Sigma) for 15 minutes at room temperature. After washed with PBS, cells were permeabilized by 0.5% Triton X‐100 (Sigma) for 15 minutes at room temperature. Cells were washed and incubated with 4% bovine serum albumin (BSA) for 1 hour at room temperature, which were then stained with primary antibodies at 4℃ overnight. These primary antibodies were used: OCT4, SOX2, SSEA4, TRA‐1‐81, AFP, SMA, NESTIN (1:200; Abcam). We washed the cells and incubated them with the secondary antibodies for 1 hour at room temperature, which were as following: Alexa Fluor 488 Goat anti‐Mouse IgG (H + L; 1:500; Invitrogen) and Alexa Fluor 594 Donkey anti‐Rabbit IgG (H + L; 1:500; Invitrogen). The cells were washed and Nuclei were stained with DAPI (1 μg/mL; Life Technology) for 10 minutes. The stained cells were observed through a confocal microscope (Nikon). Alkaline phosphatase (AP) staining was performed by Alkaline Phosphatase Assay Kit (Beyotime) according to the manufacturer's instruction, and cells were analysed via a microscope (Leica).

### 2.5 Differentiation of three germ layers and teratoma formation

The iPSCs were treated with dispase and cultured in ultra‐low attachment 6‐well plate with the medium consisting of 1× Knockout DMEM/F12 (Gibco), 20% FBS (BI), 2 mmol/L GlutaMAX‐I (Gibco), 0.1 mmol/L NEAA (Gibco), 0.05 mmol/L β‐ME (Invitrogen), 50 U/mL P/S (Gibco). After 7 days, embryonic bodies (EBs) were formed and adherent culture was used with the medium containing 1× Knockout DMEM/F12, 10% FBS, 2 mmol/L GlutaMAX‐I, 50 U/mL P/S for more than 10 days. Then, three germ layers were formed and cells were stained. The care and experiment with mice in this study were approved by the ethics committee of the Third Affiliated Hospital of Guangzhou Medical University, and we complied with the institutional ethical guidelines for animal experiment. The iPSCs from a 60 mm dish were harvested and injected into the inguinal grooves of 6‐week‐old immunodeficient mice. The teratomas were formed after 6 weeks and taken out for fixation using 4% paraformaldehyde. After the teratomas were embedded in paraffin and stained with H&E, specimens were examined for the presence of three germ layers.

### 2.6 Karyotype analysis and the assay for short tandem repeat

The iPSCs were incubated with the culture medium added 0.25 mg/mL colcemid (Invitrogen) for 4 hours and then incubated in mixed solution containing 0.4% sodium citrate and 0.4% potassium chloride (1:1, vol/vol) at 37℃ for 5 minutes. The cells were fixed three times with a methanol/acetic acid solution (3:1, vol/vol). Subsequently, after digestion with 0.8% trypsin and Giemsa staining, cells were performed with chromosome analysis. Genomic DNA was extracted from iPSCs by DNeasy Blood & Tissue Kit (Qiagen), and the assay for short tandem repeat (STR) were performed via Promega PowerPlex 21 System Kit (Promega). Capillary electrophoresis was performed using an ABI 3100 Genetic Analyzer (Applied Biosystems).

### 2.7 T7 Endonuclease I assay

We designed different primers near the predicted gRNA off‐target sites throughout the whole genome from the online software CCTop. When using CCTop, we chose a custom target selection with in vitro transcription while the species was set as Human (Homo sapiens GRCh37/hg19). Other parameters were unchanged. These fragments of Genomic DNA extracted from iPSCs were amplified with the primers by PrimeSTAR GXL DNA Polymerase (TAKARA) and then purified through Universal DNA Purification Kit (Tiangen). The purified products were used for T7 Endonuclease I (T7E1) assay following the manufacturer's protocol (New England BioLabs) and analysed on 2% agarose gels using Gel Imaging System (Bio‐Rad).

### 2.8 Whole exome sequencing and Sanger sequencing

The exome sequencing library was established with genomic DNA samples derived from iPSCs by Agilent‐V6 System. Whole exome sequencing was performed on Illumina NovaSeq6000 sequencer with 150bp pair‐end. Contrasting to human genome Hg19, the data were analysed through Burrows‐Wheeler Aligner (BWA; v.0.7.5a). We used Verita Trekker System and EnliveN System to detect SNVs and indels (BerryGenomics). All the results of Sanger sequencing in the study were analysed by IGE company.

### 2.9 Flow cytometric analysis

Differentiated hematopoietic cells at day 12 or 22 were collected by the treatment with Accutase for 15 minutes and disposed with 40 μm filter. Cells were stained with anti‐human CD34‐APC monoclonal antibody or anti‐human CD235a‐APC monoclonal antibody (BD) in 100 μL FACS buffer consisting of PBS and 2% FBS at 4°C for 30 minutes. Then, cells were washed and resuspended by FACS buffer. Flow cytometric analysis was performed via cell sorter (BD Aria III), and data were analysed by software FlowJo.

### 2.10 Statistical analysis

The data were subjected to statistical analysis by unpaired Student's two‐tailed *t* test, which were presented as means ± SEM. A value of *P* < .05 was considered to be a statistically significant result. The editing efficiency = (the quantity of indel clone, homozygous repairing clone and heterozygous repairing clone/ the quantity of total tested clone) × 100%. The repairing efficiency = (the quantity of homozygous repairing clone and heterozygous repairing clone/ the quantity of total tested clone) × 100%.

## 3 RESULTS

### 3.1 The design of different gene correction strategies for the HBB IVS2‐654 mutation and ssODN selection

**Figure 1:**
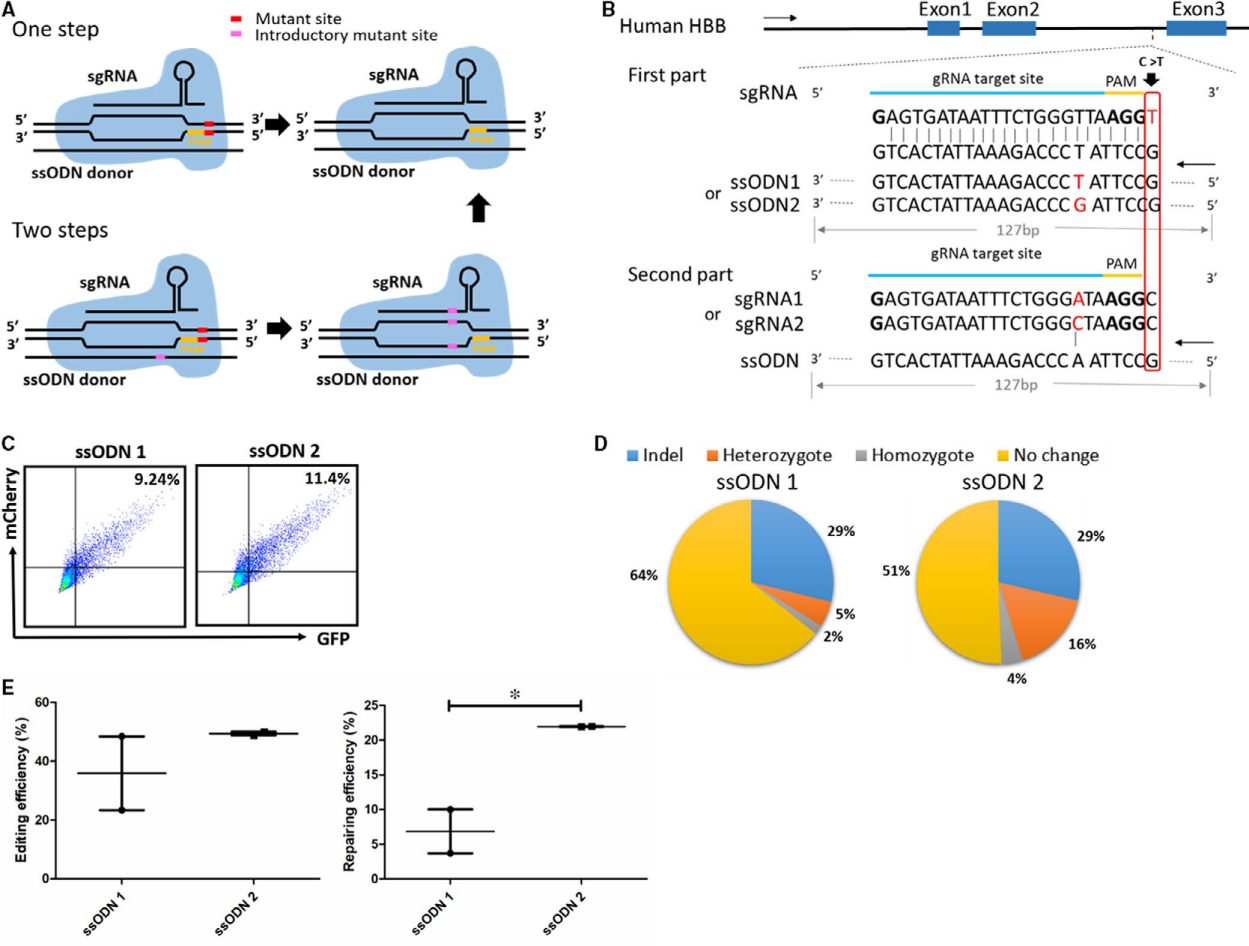
SsODN selection and single‐stranded template repair of the HBB IVS2‐654 mutation in β‐thalassaemia iPSCs. A, Schematic of different gene correction strategies for the human HBB IVS2‐654 mutation using CRISPR/Cas9 and ssODN. B, The two‐step experimental scheme to repair the HBB IVS2‐654 mutation in β‐thalassaemia iPSCs. Two pairs of gRNA and donor were designed. The nucleobase in red frame was the HBB IVS2‐654 mutation site, and the introductory mutant site was marked in red. C‐D, Evaluation of the ssODN with different introductory mutations targeting the HBB IVS2‐654 mutant site. C, Flow cytometric analysis of cotransfection efficiency of gRNA with mCherry reporter, Cas9 with GFP reporter and ssODN 1 or ssODN 2. D, Distribution of clones’ genotype from Sanger sequencing after editing the HBB IVS2‐654 mutation with different ssODNs. E, Efficiency of different ssODNs from editing and repairing. Results were represented as mean ± SEM for n = 2 individual experiments; *, *P* < .05; *t* test

According to the regulation of PAM, a 20‐bp gRNA, which is adjacent to AGG, was designed near the HBB IVS2‐654 (C > T) mutation. It was also proved to have higher efficiency in previous research.^21^ Contrasted to dsDNA, ssODN is more efficient for HDR.^19^, ^22^ So we used a 127 bp‐ssODN in the complementary strand of gRNA as the donor. However, because the IVS2‐654 mutation that the designed gRNA did not contain is in intron 2 of the HBB gene and we could not make a synonymous mutation, we made a two‐step strategy avoiding the occurrence of secondary cleavage. The first part was that we corrected the HBB IVS2‐654 mutation and introduced a new mutation belonging to the region of gRNA at the same time. For the second part, we repaired the introductory mutation. We designed two different introductory mutations. Thus, two different ssODNs were used in the first part and two unlike gRNAs were used in the second part (Figure 1A,B).

To correct the biallelic HBB IVS2‐654 mutation, CRISPR/Cas9 system and ssODN were utilized in iPSCs which derived from the reprogramming of a patient's fibroblasts. To evaluate which ssODN had higher HDR efficiency for the two‐step strategy, flow cytometric analysis was performed by gRNA with mCherry reporter, Cas9 with GFP reporter at 48 hours after the electroporation of gRNA vector, cas9 plasmid and ssODN 1 or ssODN 2 (Figure 1C). Double positive cells were harvested and re‐planted with low density. After about a week, the clones were picked up and identified via PCR. We found 29% of clones were indels in the ssODN 1 group with a total of 5% of them were heterozygote at mutant site and 2% of them were homozygote. While 29% of clones were indels, in the ssODN 2 group we found that 16% of them were heterozygote and 4% of them were homozygote (Figure 1D). The results indicated ssODN 1 and ssODN 2 had similar editing efficiency but ssODN 2’s efficiency was more stable. SsODN 2 was also more efficient than ssODN 1 for repairing. Therefore, gRNA2 and ssODN 2 were adopted in the next experiment for the two‐step strategy.

### 3.2 More efficient strategy for repairing the HBB IVS2‐654 mutation from reducing the occurrence of secondary cleavage

**Figure 2:**
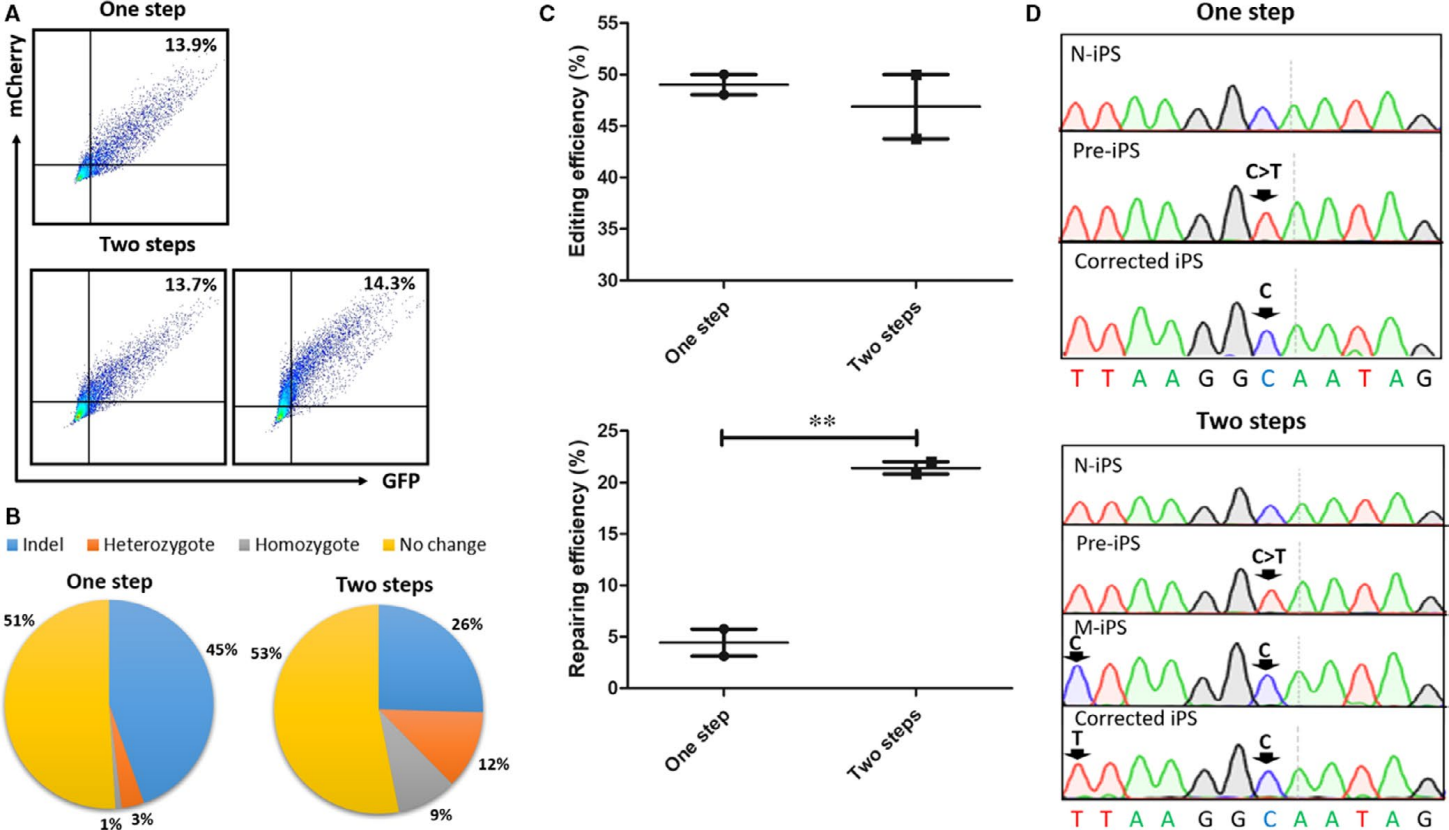
Evaluation of different gene correction strategies for the HBB IVS2‐654 mutation using CRISPR/Cas9 and ssODN. A, Flow cytometric analysis of cotransfection efficiency of gRNA with mCherry reporter, Cas9 with GFP reporter and ssODNs for different strategies. B, Distribution of clones’ genotype from Sanger sequencing after correcting the HBB IVS2‐654 mutation with different strategies. C, Efficiency of different gene correction strategies for the HBB IVS2‐654 mutation from editing and repairing. Results were represented as mean ± SEM for n = 2 individual experiments; **, *P* < .01; *t* test. D, Sanger sequencing results of C > T mutation site in iPS cells before and after gene correction with PCR product, showing homozygous correction. N‐iPS represented the normal iPS cell line. Pre‐iPS represented the iPS cell line that had the HBB IVS2‐654 mutation. M‐iPS represented an iPS cell line from the first part in two‐step strategy. Arrows showed the HBB IVS2‐654 mutant site or the introductory mutant site

Next, we compared the two strategies. As it was previously described, we electroporated gRNA vector, Cas9 plasmid and ssODN into iPSCs for the one‐step group. Notably, a two‐time electroporation was performed for the two‐step group. In the first part gRNA vector, Cas9 plasmid and ssODN 2 were utilized; while gRNA2 vector, Cas9 plasmid and ssODN were utilized for the second part. After each 48 hours, we obtained double positive cells though FACS with mCherry (gRNA) and GFP (Cas9; Figure 2A) and then cultured them with low density. Until the formation of clones, we picked up 40‐60 clones every time and screened the corrected cell lines at the mutant site via PCR. We could repair the HBB IVS2‐654 mutation by both of the two strategies and detected the introductory mutant in the first part but not in the second part for the two‐step strategy (Figure 2D). The results for the one‐step group showed 45% of clones were indels, 3% of them were heterozygote for gene editing and 1% of them were homozygote. In the two‐step group, while 26% of clones were indels, 12% of them were heterozygote and 9% of them were homozygote. As demonstrated, the editing efficiency for the second strategy did not have much difference compared to the first, whereas the repairing efficiency for the two‐step strategy was much higher than the one‐step strategy (Figure 2B,C).

### 3.3 Characterization of pluripotency in the gene‐corrected iPSCs

**Figure 3:**
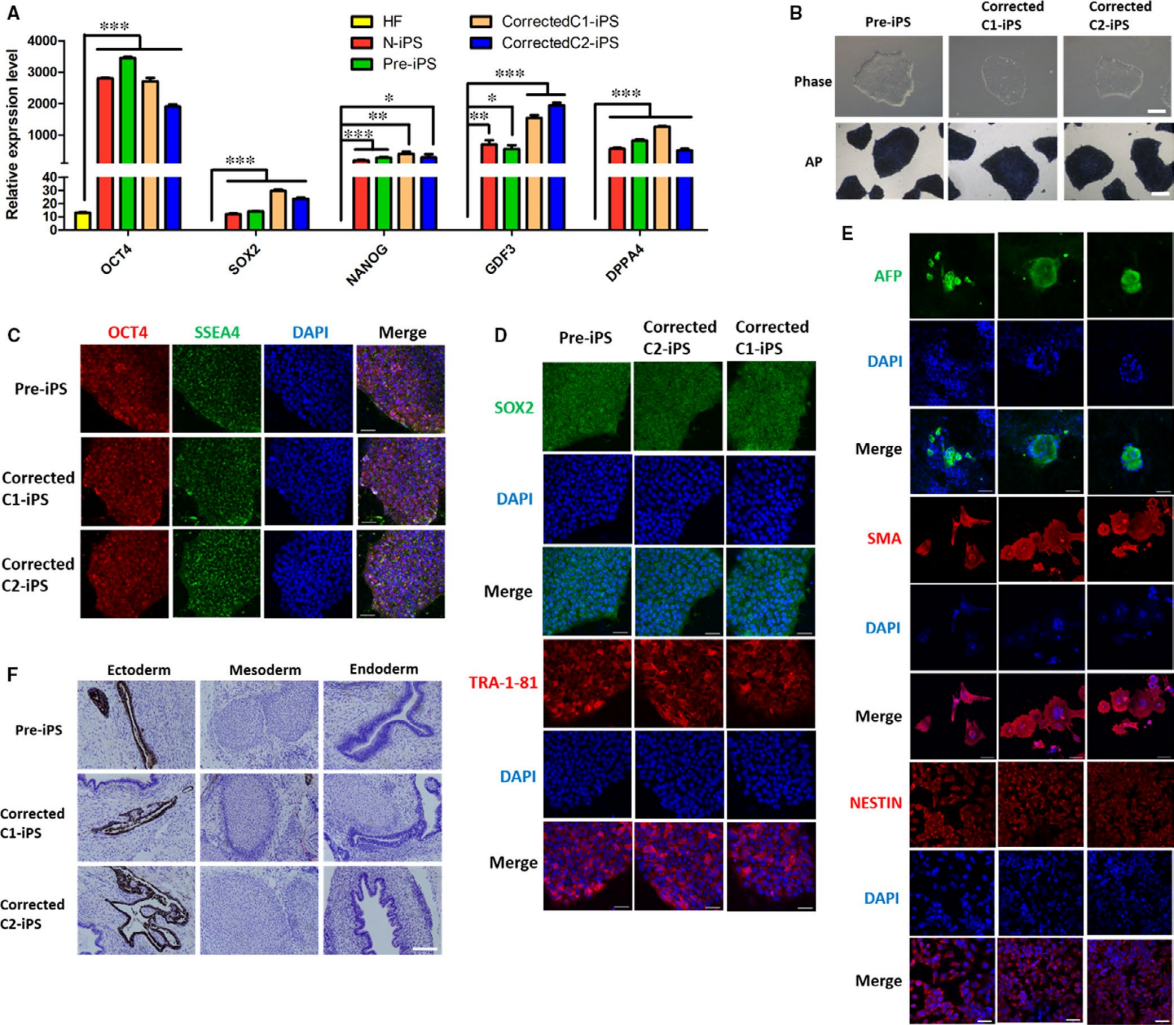
Pluripotency of gene‐corrected iPSC clones. A, Quantitative PCR analysis of genes associated with self‐renewal and pluripotency. Endogenous gene expression including OCT4, SOX2, NANOG, GDF3 and DPPA4 was measured in n‐iPS, pre‐iPS and the two corrected iPS cell lines (Corrected C1‐iPS and Corrected C2‐iPS). HF represented human fibroblast cell line. Results were represented as mean ± SEM for n = 3 individual experiments; *, *P* < .05, **, *P* < .01, ***<0.001; t test. B, Representative images of iPSC morphology and AP staining from pre‐iPS, corrected C1‐iPS and corrected C2‐iPS cell lines. Scale bar, 200 μm. C, D, Immunostaining images of pluripotency‐associated markers OCT4, SSEA4, SOX2 and TRA‐1‐81 from the three iPSC lines. Scale bar, 100 μm. E, Immunostaining for AFP (Endoderm marker), SMA (Mesoderm marker), NESTIN (Ectoderm marker) in cells after differentiation in vitro derived from EB formation of the three iPSC lines. Scale bar, 100 μm. F, Haematoxylin and eosin staining of teratomas confirming the presence of three germ layers derived from pre‐iPS, corrected C1‐iPS and corrected C2‐iPS cell lines. Scale bar, 500 μm

To identify whether the iPSCs after gene repair retain normal pluripotency, two gene‐corrected iPS cell lines (corrected C1‐iPS and corrected C2‐iPS) from the two‐step strategy were chosen for further detecting. The iPSCs before (pre‐iPS) or after gene correction displayed typical morphology and the AP staining of them was positive (Figure 3B). Quantitative PCR analysis showed the iPSCs before or after gene correction had higher expression of pluripotency‐related genes, such as *OCT4, SOX2, NANOG, GDF3* and *DPPA4*, comparing with the patient's fibroblasts (Figure 3A). Immunofluorescence results also revealed the typical pluripotency markers: OCT4, SSEA4, SOX2 and TRA‐1‐81 were expressed in these iPSCs (Figure 3C, 3). Moreover, pre‐iPS, corrected C1‐iPS and corrected C2‐iPS cell lines could differentiate into three different germ layers in vitro after the formation of EBs, which were showed via immunofluorescence (Figure 3E). We also acquired teratomas that had three different germ layers in vivo at 6 weeks after these iPSCs were individually transplanted into immunodeficient mice (Figure 3F). All the above results indicated that the gene‐corrected iPSCs kept pluripotency.

### 3.4 The stability of genome in the gene‐corrected iPSCs

**Figure 4:**
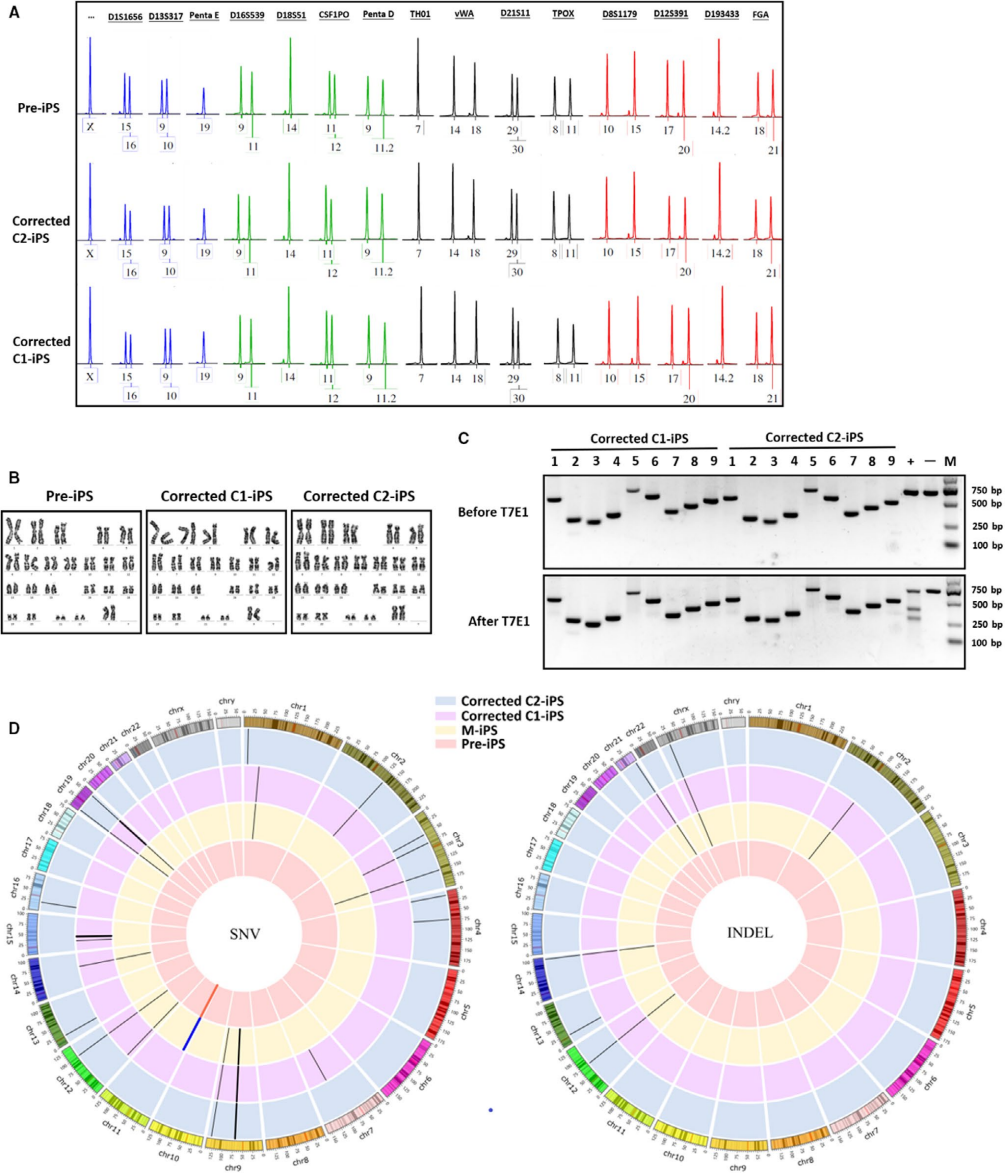
Genome stability of iPSCs after gene repair. A, STR analysis of pre‐iPS, corrected C1‐iPS and corrected C2‐iPS cell lines, confirming they derived from the same patient. B, Karyotypes analysis of pre‐iPS, corrected C1‐iPS and corrected C2‐iPS cell lines. C, T7E1 assays for PCR product of corrected C1‐iPS and corrected C2‐iPS cell lines at the 9 predicted off‐target sites. ‘−‘ was from T7E1 assay for PCR product of pre‐iPS at the HBB IVS2‐654 mutant site as a negative control. ‘+’ represented T7E1 assay for PCR product of a cell line that has a indel as a positive control. M means a DNA marker. D, The whole exome sequencing analysis of cell lines before or after gene correction. The left chart showed single‐nucleotide variations in m‐iPS, corrected C1‐iPS and corrected C2‐iPS cell lines contrasted to pre‐iPS, and the black bars represented individual SNVs. The red bar indicated the HBB IVS2‐654 mutation, and the blue bar represented the introductory mutation in two‐step strategy. The right chart showed insertions and deletions in m‐iPS, corrected C1‐iPS and corrected C2‐iPS cell lines contrasted to pre‐iPS, and the black bars represented individual indels

At first, we made sure that pre‐iPS, corrected C1‐iPS and corrected C2‐iPS derived from the same patient though STR assay (Figure 4A). These iPSCs had normal karyotype (Figure 4B). We obtained these predicted off‐target sites using the online software CCTop. Then, T7E1 assay was performed after we did PCR with the extracted DNA of iPSCs. The T7E1 assay and Sanger sequencing revealed there was no off‐target mutagenesis at top 9 sites in corrected C1‐iPS and corrected C2‐iPS cells (Figure 4C, Table S1). To further confirm whether gene editing affected the genome of corrected iPSCs except for the HBB IVS2‐654 mutation, we examined the whole exome sequencing of the gene‐corrected iPS cell lines via high‐throughput sequencing. The result showed 10 SNVs and 5 indels were in an iPS cell line (m‐iPS) from the first part in the two‐step strategy. Corrected C1‐iPS cell line had 17 SNVs and 5 indels while corrected C2‐iPS cell line had 19 SNVs and 5 indels. From the data, there were the same 10 SNVs and 4 indels in these three cell lines, which means it produced 10 SNVs and 4 indels in the first part. In the second part, 7 SNVs and no indels in the corrected C1‐iPS cell line along with 9 SNVs and 1 indel in the corrected C2‐iPS cell line appeared (Figure 4D, Table S2‐S4). All the sites of SNVs and indels were not in accord with the predicted off‐target site. The sequences at these sites had too many mismatches with gRNA which made it not easy for gRNA to target these sites. Thus, that suggested CRISPR/Cas9 system was not the direct reason for the mutagenesis.

### 3.5 The restoration of HBB gene expression in the gene‐corrected iPSCs

**Figure 5:**
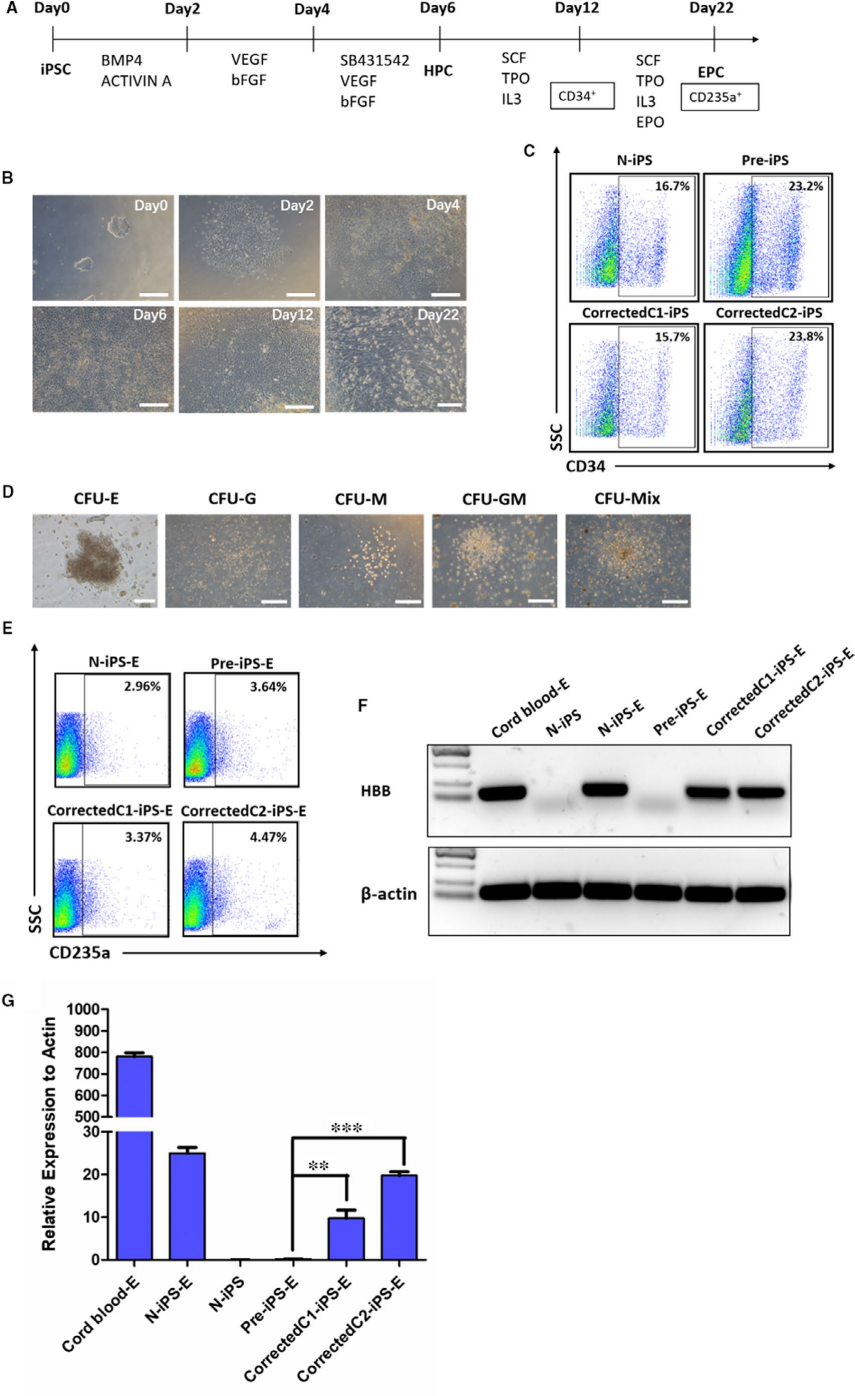
Hematopoietic differentiation of gene‐corrected iPSCs. A, Experimental scheme for a five‐step hematopoietic differentiation strategy from iPSCs. B, Images of representative morphology changes in different hematopoietic differentiation stages. Scale bar, 500 μm (Day 0, day 2, day 4, day 6, day 12); Scale bar, 200 μm (Day 22). C, Flow cytometric analysis of CD34^+^ expression at day 6 during the hematopoietic differentiation of n‐iPS, pre‐iPS, corrected C1‐iPS and corrected C2‐iPS. D, Representative images of colony morphologies for CFU assay after another 14 days differentiation using the CD34^+^ cells at day 12 during the hematopoietic differentiation. Scale bar, 100 μm (CFU‐E); Scale bar, 500 μm (CFU‐G, CFU‐M, CFU‐GM, CFU‐MIX). E, Flow cytometric analysis of CD235a^+^ expression at day 22 during the hematopoietic differentiation of n‐iPS, pre‐iPS, corrected C1‐iPS and corrected C2‐iPS. F, The agarose gel images of RT‐PCR product by amplifying HBB cDNA of CD235a^+^ cells derived from the hematopoietic differentiation of n‐iPS, pre‐iPS, corrected C1‐iPS and corrected C2‐iPS cell lines. CD34^+^ cells from cord blood were used as a positive control (Cord blood‐E), and normal iPSCs were used as a negative control. G, Quantitative PCR analysis of HBB gene expression (normalized to β‐actin) in CD235a^+^ cells derived from the hematopoietic differentiation of cell lines before or after gene correction. Results were represented as mean ± SEM for n = 3 individual experiments; **, *P* < .01, ***<0.001; *t* test

The patients with β‐thalassaemia show issues related to the β‐globin chain synthesis. To investigate the expression of HBB gene and the functional effect of gene correction, iPSCs were differentiated into hematopoietic progenitor cells (HPCs) and then erythroid precursor cells (EPCs) using previously established protocols.^20^ Through a 22‐day monolayer culture, iPSCs expanded undergoing five steps with morphological changes (Figure 5A,B). After inducing differentiation, iPSCs changed to be endothelial‐like cells and we began to acquire HPCs at day 6. At day 12, FSCs analysis showed all of them could express the marker CD34. The CD34^+^ cells were sorted and cultured in semisolid methylcellulose medium for CFU assay (Figure 5C). Five different kinds of clones formed after 2 weeks, confirming iPSCs after HBB gene correction had the ability to differentiate into various blood lineages (Figure 5D). At day 22 of the hematopoietic differentiation, EPCs were obtained from sorting cells via flow cytometric analysis that was performed with the marker CD235a (Figure 5E). Then, we designed a F‐primer in Exon 2 and a R‐primer in the spliced junction of Exon 2 and Exon 3. Due to RT‐PCR assay amplifying the HBB cDNA from these CD235a positive cells, as well as quantitative PCR analysis showing the expression of HBB gene in CD235a positive cells from corrected C1‐iPS and corrected C2‐iPS cell lines were far higher than pre‐iPS cell line, we concluded the function of HBB gene was successfully restored in the gene‐corrected iPSCs (Figure 5F,G).

## 4 DISCUSSION

Thalassaemia is one of the most common genetic disease resulting from the imbalance of globin chain production mostly caused by gene mutation.^23^ Due to the limitations of different clinical treatment at present, patients cannot have effective recovery, which emphasizes the importance of seeking new ways for therapy that targets thalassaemia. Hope is offered and found with engineered nucleases. Zinc‐finger nucleases (ZFNs) and transcription activator‐like effector nucleases (TALENs) are produced via proteins fused with the nuclease domain of the restriction enzyme FokI and they work through protein‐DNA interaction.^24^ Previous reports have shown HDR rates varied at 33% and 68% when we used TALEN and dsDNA to edit. They are not used widely because they need to engineer and clone a new protein when targeting a new site, which is a complicated process. The difference of the repairing efficiency is large as well.^21^, ^25^ However, CRISPR/Cas9, an RNA‐guided system, is easier to be designed and constructed with lower costs. Gene correction is based on the sequence‐specific targeting of gRNA, the DSB caused by Cas9 protein and the repair of gene via the donor template for HDR. In previous report, the HBB IVS2‐654 mutation in β‐thalassaemia iPSCs had been corrected by CRISPR/Cas9 with dsDNA.^21^ Another team produced the disruption of genomic elements to make indels for removing the mutation using LbCas12a RNP, and the efficiency was up to 76.6%. Whether there are potential risks for changing genome sequences is unknown.^26^ Nevertheless, contrasted to dsDNA, ssODN is more efficient for HDR and has lower cytotoxicity.^19^ In this study, we demonstrated an efficient approach for correcting the biallelic HBB IVS2‐654 mutation in β‐thalassaemia iPSCs combining CRISPR/Cas9 system with ssODN which were electroporated into iPSCs. Because antibiotic selection may interfere with the expression of corrected gene,^27^, ^28^ the positive cells with fluorescent reporter were harvested by FACs. We finally acquired the corrected iPS cell line after Sanger sequencing for expanding clones. Next, many assays revealed the corrected iPSCs remained pluripotency, genome stability and differentiation ability. Most importantly, we examined the expression of HBB gene and concluded the function of HBB gene was successfully restored in the gene‐corrected iPSCs.

Because most patients who have the heterozygous HBB IVS2‐654 mutation do not show to have symptoms, repairing the monoallelic mutation can cure them. In the one‐step group, the repairing efficiency was 4% on average including both heterozygote and homozygote. However, for that strategy, we could not design gRNA including HBB IVS2‐654 mutant site, which should be adjacent to NGG due to spCas9. Therefore, gRNA was designed near the mutation. Then, there is the problem that gRNA can still target the sequence after gene correction. The mutation is from an intron and we cannot make a synonymous mutation. To reduce the occurrence of secondary cleavage, we adopted a two‐step strategy. In the first part, we corrected the HBB IVS2‐654 mutation and introduced a new mutation at the same time which belong to the region of gRNA. Next, we repaired the introductory mutation in the second part. Two strategies had similar editing efficiency, whereas the repairing efficiency of the two‐step strategy could reach 21% on average, which was about 5 times that of the one‐step strategy. The HDR rate was also higher than it was with CRISPR/Cas9 and dsDNA in the previous report (12.3%).^21^ That revealed the two‐step strategy about inducing a new mutation can indeed reduce the occurrence of secondary cleavage and improve the repairing efficiency (Figure 2). In addition, we found that the repairing efficiency, not the editing efficiency, also has significant difference if inducing a new mutation from different nucleotide (Figure 1). Nevertheless, comparatively speaking, the two‐step strategy is not simple. Hence, we tried another method in the one‐step method. We electroporated all the gRNAs and ssODNs as well as Cas9 used in the two‐step strategy into iPSCs together in a single time. We assumed that they could work in cells twice because plasmids can stay in cells for several days. But the final result showed the editing efficiency from the mixed gRNAs and ssODNs was lower than that of the one‐step strategy mentioned above (Figure S1). We think the most likely reason is that the mass of gRNA and gRNA2 were comparing half to the mass of gRNA used in the one‐step strategy because the same total mass of gRNA should be kept in the experiment. For this aspect, we can solve the problem by utilizing a vector which can carry multiple gRNAs in the further study. Moreover, there are many other ways to improve the efficiency of gene repair, for example, adding small molecules, synchronizing the cell cycle, and adjusting delivery timing and methods.^29^, ^30^, ^31^ Besides, using ribonucleoprotein (RNP) delivery of Cas9 with gRNAs consistently can increase activity in cells and then enhance the efficiency.^19^, ^31^

Since gRNA can target similar sequences in a genome, it is possible to have off‐targets when we use SpCas9 system.^32^, ^33^ Thus, we did T7E1 assay for 9 potential off‐target site analysed by the online software CCTop and confirmed them by Sanger sequencing. This revealed that the corrected iPS cell line had no indel. Next, the whole exome sequencing was performed to assess off‐targeting effects, and then, we found some SNVs and indels from the corrected iPS cell lines contrasting to the iPS cell line before repair. But all the sites from these SNVs and indels were not in the potential off‐target regions according to gRNA targeting sequence (Figure 4, Tables S2‐S4). We considered the fact that sometimes high‐throughput sequencing can have false‐positive results, and it is possible for cells to produce some mutations after more and more passage. To solve these problems, we can do experiments with cells at the early passages and perform Sanger sequencing to identify the results from the whole exome sequencing. To improve SpCas9 system's targeting specificity, we also can adopt the following strategies: shorter gRNAs design or gRNAs with two unpaired Gs on the 5′ end which are more sensitive to mismatches,^34^, ^35^ paired nCas9s,^36^, ^37^ paired dCas9‐FokI nucleases.^38^, ^39^ What should be noticed is that they may lower the efficiency although greatly enhancing the specificity. Nonetheless, no matter which strategy to be chosen, the whole exome sequencing needs to be performed before clinical application.

IPSCs, reported initially by Yamanaka, who won the Nobel Prize for Physiology or Medicine in 2012, have the property of self‐renewal and pluripotency, which offers a promise for regenerative medicine.^40^ With great advances in iPSC technology, many researchers have used it for clinical trials. A Japanese team differentiated autologous iPSCs into retinal cells and transplanted them into six patients of neovascular age‐related macular degeneration. One year later, one of their cases was reported to be successful.^41^ Furthermore, Japanese teams have gone on to carry out many clinical trials for medical issues such as heart disease, blood disease, spinal cord injury and Parkinson's disease. In this study, we repaired the HBB IVS2‐654 mutation in β‐thalassaemia iPSCs with CRISPR/Cas9 system and ssODN. The great potential of iPSCs make us believe that utilizing them for gene correction can provide preclinical study for autologous cell therapy. Recent research demonstrated that combining CRISPR/Cas9 technology with rAAV6 can target human hematopoietic stem cells (HSCs) for gene repair via HDR, which offers an efficient technology for autologous cell therapy of β‐Thalassaemia.^42^, ^43^ Moreover, iPSCs have the ability to differentiate into all cell types. Thus, we did hematopoietic differentiation and found that the function of HBB gene was successfully restored in the gene‐corrected iPSCs (Figure 5).

## 5 CONCLUSIONS

From this study, we describe a one‐step approach to correct the biallelic HBB IVS2‐654 mutation in β‐thalassaemia iPSCs through CRISPR/Cas9 and ssODN‐mediated HDR. For the mutation in an intron and no appropriate gRNA containing the mutant site, a two‐step strategy can be adopted to reduce the occurrence of secondary cleavage for improving the repairing efficiency. The corrected iPSCs keep pluripotency and genome stability. Moreover, the expression of HBB gene can be restored in vitro after hematopoietic differentiation. Therefore, our findings demonstrate that the strategies of gene correction we report here will facilitate the development of cell therapy for genetic disease in iPSCs.

## CONFLICT OF INTEREST

The authors declare no competing interests.

## AUTHOR CONTRIBUTIONS

XZY: involved in conception and design, collection and assembly of data, data analysis and interpretation, manuscript writing; XYJ, Y.Y, W.D: involved in data analysis and interpretation; LSH, XYT, CDY, L.D, HLN, S.B, YYH: collected and assembled the data; SXF: involved in conception and design, provision of study materials or patients, financial support, manuscript review. All authors read and approved the final version of the manuscript.

## FUNDING INFORMATION

National Natural Science Foundation of China, Grant/Award Number: 31872800, 31801124, 31701299, 31701288; Foundation of Guangzhou Science and Technology Commission, Grant/Award Number: 201803040009; Natural Science Foundation of Guangdong Province, Grant/Award Number: 2014A030312012; Guangdong Province Science and Technology Project, Grant/Award Number: 2016B030229008; China Postdoctoral Science Foundation, Grant/Award Number: 2018M633029, 2018T110858, 2018M64077.

## Data Availability

The raw data of whole exome sequencing reported in this paper have been deposited in the Genome Sequence Archive in BIG Data Center, Beijing Institute of Genomics (BIG), Chinese Academy of Sciences, under accession numbers CRA001893, CRA001893 that are publicly accessible at https://bigd.big.ac.cn/gsa.
