# Supplementary material for: Efficient gene correction of an aberrant splice site in β‐thalassaemia iPSCs by CRISPR/Cas9 and single‐strand oligodeoxynucleotides
Source: J Cell Mol Med. 2019 Oct 21;23(12):8046–57. doi: 10.1111/jcmm.14669 (PMC6850948; doi:10.1111/jcmm.14669)
Supplement: Supplementary file 1 [file JCMM-23-8046-s001.pdf]

Supplementary material 1

Efficient gene correction of an aberrant splice site in β-Thalassemia induced pluripotent stem cells by CRISPR/Cas9 and Single-strand oligodeoxynucleotides

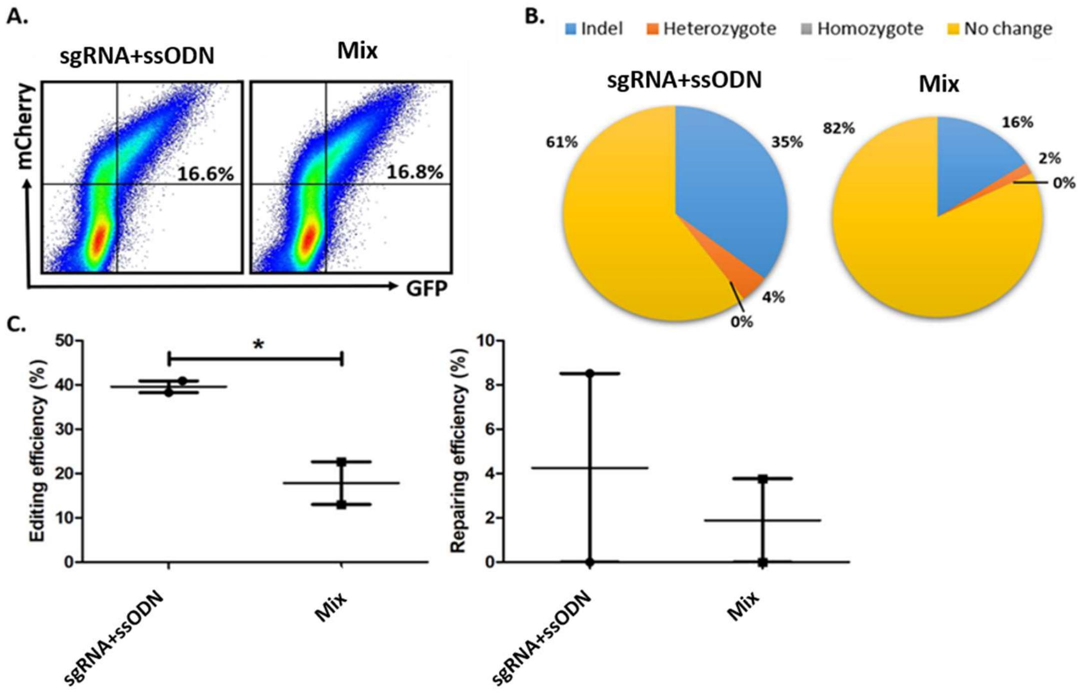

SUPPLEMENTARY FIGURE 1 Lower efficiency for the strategy of the mixed gRNAs and ssODNs. (A) Flow cytometric analysis of co-transfection efficiency of gRNA with mCherry reporter, Cas9 with GFP reporter and ssODNs for different strategies. (B) Distribution of clones' genotype from sanger sequencing after correcting the HBB IVS2-654 mutation with different strategies. (C) Efficiency of different gene correction strategies for the HBB IVS2-654 mutation according to editing and repairing. Results were represented as mean ± SEM for n=2 individual experiments; *, $p<0.05$; t-test.

Supplementary table 1 The information about the 9 predicted gRNA off-target sites using the online software CCTop.

	Target sequence	PAM	MM	Gene	Coordinates
	CAGTGATAATTTCTGGGTTA	AGG	0	HBB	chr11:5225924-5225946
1	CAGTGATTATTTCTGGGTTA	TGG	1	HHAT	chr1:210646655-210646677
2	GAGTGATGATTTCTGGGTTA	AGG	2	ATXN10	chr22:45722099-45722121
3	GAATGATACTTTCTGGGTTA	GGG	3	CNTNAP2	chr7:146029914-146029936
4	GAGTGGTTATTTCTGGGTTA	TGG	3	RP11-482E14.1	chr10:3538305-3538327
5	CATTTTGAATTTCTGGGTTA	GGG	4	NCKAP5	chr2:133936858-133936880
6	AAGGAATATTTTCTGGGTTA	GGG	4	KANSL1	chr17:44156490-44156512
7	CAGGCAAAATCTCTGGGTTA	AGG	4	SLC8A1	chr2:40748020-40748042
8	CTATGATAATTTCTTGGTTA	GGG	3	CCDC178	chr4:142158789-142158811
9	CAGTAATGTTCTCTGGGTTA	AGG	4	AF131216.6	chr8:11176897-11176919

Supplementary table 2 The information about single-nucleotide variations in a cell line from the first part and two corrected iPS cell lines contrasted to the cell line before gene repair by The whole exome sequencing.

Simple	Coordinates	SNV	Gene	Genotype
M, C1, C2	chr12: 100784835	C>A	SLC17A8	Heterozygote
M, C1, C2	chr19: 51988321	G>C	PLIN5	Heterozygote
M, C1, C2	chr19: 4529193	G>A	SIGLEC8;SIGLEC12	Heterozygote
M, C1, C2	chr3: 151164060	T>C	IGSF10	Heterozygote
M, C1, C2	chr9： 130477905	C>T	PTRH1	Heterozygote
M, C1, C2	chr9： 69420348	G>A	ANKRD20A4	Heterozygote
M, C1, C2	chr9： 69423721	G>A	ANKRD20A4	Heterozygote
M, C1	chr1： 46499807	G>T	MAST2	Heterozygote
M, C1	chr12： 11420626	G>T	PRB3	Heterozygote
M, C1	chr14： 65260078	C>T	SPTB	Heterozygote
C1, C2	chr3： 75786381	T>C	ZNF717	Heterozygote
C1	chr15： 30382038	A>G	TJP1;ULK4P3	Heterozygote
C1	chr15： 41688915	C>T	NDUFAF1	Heterozygote
C1	chr15： 43924420	C>T	CATSPER2	Heterozygote
C1	chr19: 50249950	C>T	TSKS	Heterozygote
C1	chr2 : 130899940	T>C	CCDC74B	Heterozygote
C1	chr7: 100417328	G>A	EPHB4	Heterozygote
C2	chr3: 75781272	C>A	LINC00960;ZNF717	Heterozygote
C2	chr13: 20066994	T>C	TPTE2	Heterozygote
C2	chr16 :21747639	G>T	OTOA	Heterozygote
C2	chr3: 52417902	G>A	DNAH1	Heterozygote
C2	chr4: 11401177	C>A	HS3ST1	Heterozygote
C2	chr2: 132290441	G>A	CCDC74A	Heterozygote
C2	chr1: 10521671	C>T	DFFA	Heterozygote
C2	Chr4： 74308122	G>T	AFP	Heterozygote

M: M-iPS; C1: corrected C1-iPS; C2: corrected C2-iPS.

Supplementary table 3 The information about insertions and deletions in a cell line from the first part and two corrected iPS cell lines contrasted to the cell line before gene repair by The whole exome sequencing.

Simple	Coordinates	Reference	Alteration	Gene	Genotype
1-M, C1, C2	chr12: 75900343	-	T	KRR1	Heterozygote
2-M, C1, C2	chrX: 24382501	-	CTGCTCCTG<br>CTC	SUPT20HL1	Heterozygote
3-M, C1, C2	chr14: 102551276	TTT	-	HSP90AA1	Heterozygote
4-M, C1, C2	chr21: 42830693	CTGTCCA<br>GCCCCGT<br>AGACGTG<br>CACGCAC<br>A	-	MX1	Heterozygote
5-M, C1,	chr2：96519559	TCGT	-	TRIM43;FAHD2CP	Heterozygote
6-C2	chr13：21729952	-	GGAG	SKA3	Heterozygote

M: M-iPS; C1: corrected C1-iPS; C2: corrected C2-iPS.

Supplementary table 4 The sequence at these insertion and deletion sites in a cell line from the first part and two corrected iPS cell lines contrasted to the cell line before gene repair by The whole exome sequencing.

Simple	Coordinates	Sequence
	gRNA:	CAGTGATAATTTCTGGGTTAAGG
	gRNA2:	CAGTGATAATTTCTGGGCTAAGG
1	chr12: 75900343	TATTCCTTACTAAAGAACCTTATTTTAATGATGTCACATGCA
2	chrX: 24382501	CTCCTGCTCTAGCTGCTGCTCTGCTCCTGCTCGCTGCTCCTGCTCC<br>TGCTCCT
3	chr14: 102551276	TCTTTTTCTTTTTCTTCTTC~~TTT~~GTCTTCCTTTTCTTCAGC
4	chr21: 42830693	GTTCCCCGGTTAACCACACT~~CTGTCCAGCCCCGTAGACGTGCACG<br>CACA~~
5	chr2：96519559	AGCAAAGCTTTTGTTGCTGA~~TCGT~~TTGTTTAAGACATCATC
6	chr13：21729952	AGTTGTATTTTGATAAAAGCGGAGTAAAAAAAAAAAAGGAAATTC

Supplementary table 5 The information about primers

Primer Name	Sequence (5’ to 3’)
gRNA	GAGTGATAATTTCTGGGTTA
gRNA1	GAGTGATAATTTCTGGGATA
gRNA2	GAGTGATAATTTCTGGGCTA
ssODN	TGTAGCTGCTATTAGCAATATGAAACCTCTTACATCAGTTACAATTT<br>ATATGCAGAAATATTTATATGCAGAAATATTGCTATTGCCTTAACCC<br>AGAAATTATCACTGTTATTCTTTAGAATGGTGC
ssODN1	TGTAGCTGCTATTAGCAATATGAAACCTCTTACATCAGTTACAATTT<br>ATATGCAGAAATATTTATATGCAGAAATATTGCTATTGCCTTATCCCA<br>GAAATTATCACTGTTATTCTTTAGAATGGTGC

Primer Name	Sequence (5' to 3')
ssODN2	TGTAGCTGCTATTAGCAATATGAAACCTCTTACATCAGTTACAATTT ATATGCAGAAATATTTATATGCAGAAATATTGCTATTGCCTTAGCCC AGAAATTATCACTGTTATTCTTTAGAATGGTGC
HBB	F: TGCATCAGTGTGGAAGTCTCA
	R: AGGAGCTGTGGGAGGAAGAT
HBB-RT	F: CTCATGGCAAGAAAGTGCTC
	R: GTTGCCCAGGAGCCTGAA
β-actin-RT	F: CGCGAGAAGATGCCCAGATC
	R: TCACCGGAGTCCATCACGA
OCT4-RT	F: GCTCTCCCATGCATTCAAACTG
	R: TCCCAAAAACCCTGGCACAAAC
SOX2-RT	F: ATCCACACTCACGCAAAAACCG
	R: AACTTCCTGCAAAGCTCCTACC
Nanog-RT	F: CAGCCCCGATTCTTCCACCAGTCCC
	R: CGGAAGATTCCCAGTCGGGTTCACC
GDF3-RT	F: CTTATGCTACGTAAAGGAGCTGGG
	R: GTGCCAACCCAGGTCCCGGAAGTT
DPPA4-RT	F: CAGAGAAGTCGAGGGAAGAGGA
	R: ACAGGTGGCAGTTTAGAAGGTA
HHAT	F: TGTTCTCACCACTGTGTGCT
	R: TTGGAATAGCTGGGGATGCT
ATXN10	F: TTATGGCAGCATGGCTCTGT
	R: TCCCTACCAGACACTCCACC
CNTNAP2	F: TCGTTAATACAGGCTTCCATTGTCT
	R: AATCCCTCTGGGAGCACTTTAAT
RP11-482E14.1	F: GGTACAGGAACGGGGTTCAG
	R: GGGCAAATCATTACCCCGGA
KANSL1	F: GATGTTTCCTGGGTGGGCTT
	R: AGGGCACACCCTCTTTGTTC
CCDC178	F: AACATTCCCAGTCTGGCCTC
	R: CAACTGCCTGCACTTTTTGGA
NCKAP5	F: CCATGCTACGATTGAGCCCAC
	R: CACTGCACTCTAGGCTGGGT
SLC8A1	F: CTCCCTCCCTGCATCAAGTTCTC
	R: TCCTTCAAGTTGTCAGATTGCCCG
AF131216.6	F: TCCAAGAAGCTGGCAGGAGG
	R: CGGATGATGGGTGCACCAGA
